# COL12A1 rs970547 Polymorphism Does Not Alter Susceptibility to Anterior Cruciate Ligament Rupture: A Meta-Analysis

**DOI:** 10.3389/fgene.2021.665861

**Published:** 2021-08-10

**Authors:** Zheng-tao Lv, Wei Wang, Dong-ming Zhao, Jun-ming Huang

**Affiliations:** ^1^Department of Orthopedics, Tongji Hospital, Tongji Medical College, Huazhong University of Science and Technology, Wuhan, China; ^2^Department of Orthopedics, Zhongshan Hospital, Fudan University, Shanghai, China

**Keywords:** COL12A1, rs970547, anterior cruciate ligament rupture, meta-analysis, collagen XII

## Abstract

**Objective:** Currently available evidence regarding the association between collagen type XII α1 (COL12A1) polymorphism and risk of anterior cruciate ligament rupture (ACLR) remains elusive. The aim of our present study was to assess the association between COL12A1 rs970547 polymorphism and ACLR risk.

**Methods:** Five online databases, namely, PubMed, EMBASE, ISI Web of Science, CENTRAL, and CNKI, were searched from their inception data up to December 2020 to identify relative observational studies. The methodological quality of each individual study was evaluated using the Newcastle-Ottawa Scale (NOS). The “model-free approach” was employed to estimate the magnitude of effect of COL12A1 rs970547 polymorphism on ACLR, and the association was expressed using odds ratio (OR) and its associated 95% confidence interval (95% CI). Subgroup analysis was performed by ethnicity and sex of included subjects.

**Results:** Eight studies involving 1,477 subjects with ACLR and 100,439 healthy controls were finally included in our study. The methodological quality of included studies was deemed moderate to high based on NOS scores. The “model-free” approach suggested no genotype differences between ACLR and healthy control for the rs970547 polymorphism, but we still used the allele model to present the combined data. Under the random-effect model, there was no significant difference in the frequency of effecting allele between ACLR and control (OR: 0.91, 95% CI 0.77, 1.08; *p* = 0.28). Stratified analysis by sex and ethnicity also showed no difference in allele frequency.

**Conclusion:** The findings of this current meta-analysis suggested that rs970547 was not associated with ACLR risk in male, female, and the overall population among Asians or Caucasians.

## Introduction

The anterior cruciate ligament (ACL) is the primary stabilizer in the human knee joint, which acts by antagonizing anterior translation of the tibia on the femur and counteracting rotational and valgus stresses. With the popularity and extensive development of national sports, the incidence of the anterior cruciate ligament rupture (ACLR) is in an increasing trend. According to epidemiological reports, ACLR accounts for as much as 50% of all knee injuries and some authors made an estimation of 250,000 ACL injuries per year worldwide (van der Hart et al., 2008; Flagg et al., 2019).

Till this moment, there is still a wide gap in understanding the detailed mechanisms of ACLR and we only confirmed that the pathogenesis of ACLR is associated with multiple factors. Although mechanical factors to the knee, such as physical activity and the type of playing surface, is accepted as an essential prerequisite for ACLR, other risk factors including sex, familial predisposition, and genetic component also predispose individuals to ACLR (John et al., 2016a). Initially, familial studies demonstrated that the risk of ACLR is more than doubled in individuals with a first-degree relative history of ACLR, suggesting the critical role of genetic factor in ACLR (Flynn et al., 2005). As we have known, ACL is a dense fibrous connective tissue, which is composed of numerous collagen fibers, such as collagen types I, III–VI, XII, and XIV, arranged in a hierarchical pattern. Collagen is the major component of ligaments, accounting for 70–80% of dry weight, and the change of ligament strength by alteration in its main components could lead to an increase in the incidence of ACLR. Type I collagen is the major fibrillar collagen and the structural stability supporter in ACL. Type V collagen is also known to be fibrillar in structure with minor quantity. Type I collagen and type V collagen, together with type III collagen, constitute the heterotypic collagen fibrils, which are the basic functional units of ligaments (Frank, 2004). The gene COL1A1, COL3A1, and COL5A1 respectively encode the α1 chains of type I, type III, and type V collagen. As a matter of fact, several studies have identified the relationship between polymorphisms within these genes and risk of ACLR (Khoschnau et al., 2008; Posthumus et al., 2009; Ficek et al., 2013; O'Connell et al., 2015; Stepien-Slodkowska et al., 2015).

**Figure 1:**
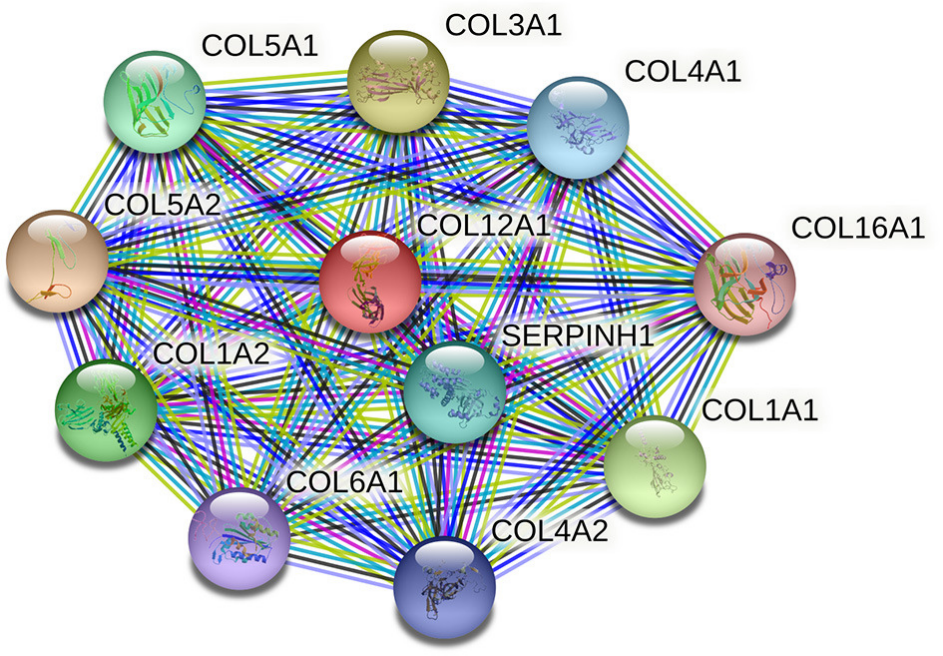
The protein–protein interaction (PPI) network of COL12A1 and its closest functional neighbors. The PPI network is generated using STRING (available at http://string-db/org). Network nodes represent proteins and the edges indicate both functional and physical protein associations.

Unlike the fibrillar collagens, type XII collagen belongs to a family of non-fibrillar collagens referred to as fibril-associated collagens with interrupted triple helices (FACITs). In immunoelectron microscopy, collagen XII is associated with the surface of collagen fibrils where it is able to form interfibrillar connections and mediate fibril interaction with other extracellular and cell surface molecules within tendons and other tissues (September et al., 2008). These fibril interactions further influence fibril and matrix density and collagenase treatment of collagen XII largely abolished incorporation into collagen I fibrils, which suggests that type XII collagen is involved in fibrillogenesis (Shaw and Olsen, 1991; Young et al., 2002; Chiquet et al., 2014). Collagen XII is a homotrimer consisting of three α1 (XII) chains, which is encoded by COL12A1 mapped to chromosome 6q12–q13. It has been shown that collagen XII is expressed in developing tendons and ligaments and both the dominant and recessive mutations in COL12A1 will lead to excessive weakness at birth, strikingly hypermobile distal joints, and absence of deep tendon reflexes (Izu et al., 2020). The protein–protein interaction (PPI) network of COL12A1 and its closest functional neighbors is illustrated in Figure 1. It shows that several proteins that closely interact with COL12A1 are also involved in the pathogenesis of ACLR, such as COL1A1, COL5A1, and COL3A1.

According to the database supported by the National Center for Biotechnology and Information, five single-nucleotide polymorphisms (SNPs) in exonic regions of COL12A1 have been identified and two SNPs (rs240736 and rs970547) are considered as non-synonymous SNPs, indicating that amino acid sequence could possibly change due to these two mutations. Up to now, several studies focusing on rs970547 SNP and the susceptibility of ACLR have been reported and only few studies have been featured to assess the association between rs240736 polymorphism and risk of ACLR. However, the results were mixed and inconclusive due to clinical heterogeneity, different ethnic populations or sexes, and small sample sizes. To overcome the limitations of these studies for elucidating the genetic roles for rs970547 SNP in the risk of ACLR, we performed a comprehensive meta-analysis of identified studies to evaluate the contradictory results from these relevant studies and to clarify the associations between rs970547 SNP in COL12A1 gene and ACLR susceptibility.

## Methods

This meta-analysis was conducted on the basis of the PRISMA guideline (Moher et al., 2009).

### Search Strategy

A systematic literature search was performed using five online databases, namely, PubMed, EMBASE, ISI Web of Science, CENTRAL, and CNKI, to identify observational studies that assessed the relationship between COL12A1 polymorphism and risk of ACLR. No language restriction was imposed on the literature search; studies published before December 2020 were searched. The search strings for English databases were as follows: “COL12A1 or collagen type XII α1 or rs970547” and “polymorphism or variant or single nucleotide polymorphism or SNP or SNPs” and “anterior cruciate ligament rupture or ACLR or ACL or anterior cruciate ligament tear or anterior cruciate ligament injury.” For Chinese database, we used “Qian Jiao Cha Ren Dai” and “COL12A1” and “Duo Tai Xing” to retrieve relative studies. Effort was also made to avoid missing Genome-wide Association Study (GWAS) that did not use COL12A1 as part of keywords or abstract; we also searched the GWAS Catalog (www.ebi.ac.uk/gwas/ last accessed on December 2020). The reference lists of relative systematic reviews and meta-analyses were also checked by two independent reviewers for additional relative studies.

### Eligibility Criteria

The inclusion criteria were made following the PICOS (participants, intervention, control, outcome, study type) principle: (i) cases were patients that surgically confirmed with ACLR; (ii) the variant of interest in our current study was rs970547 in COL12A1 gene; (iii) the control subjects were healthy and unrelated individuals without any self-reported history of ACL injury; (iv) the only outcome of our study was the association between COL12A1 polymorphism and risk of ACLR, as indicated by crude odds ratio (OR) and its associated 95% confidence interval (95% CI). The ORs could be either reported in each included study or calculated based on the genotypes distribution in ACLR and healthy control groups; (v) study type was observational study on humans (case–control study or cross-sectional study).

On the other hand, studies would be excluded if they met the following exclusion criteria: (i) animal studies and cellular experiments; (ii) reviews, editorials, case series, or case report. In case that several studies reported overlapping data, only the most comprehensive one would be considered for inclusion.

### Data Extraction

Data were extracted using a predetermined data collection sheet that included the surname of the first author, year of publication, country of origin, ethnicity of enrolled subjects, number of subjects in ACLR and control group, genotyping method used in each study, genotype counts in ACLR groups and control groups, and Hardy–Weinberg Equilibrium (HWE) results for control subjects. Discrepancies occurred during the data collection process were resolved through discussion between two investigators (ZL and WW). Otherwise, the third investigator (JH) would be consulted for his opinion.

### Methodological Quality Assessment

Two reviewers (ZL and WW) independently assessed the methodological quality of each individual study using the Newcastle-Ottawa Scale (NOS) (Wells et al., 2020). Higher scores suggested better methodological quality of included studies. Studies achieving a score lower than four stars would be excluded for analysis due to poor methodological quality. Two reviewers independently assessed the quality of each included study, and the results were compared afterwards. Any disagreement between two investigators were settled by discussion until a mutual consensus was reached.

### Statistical Analysis

Departure from HWE was evaluated by using Chi-square test to test the goodness of fit in control subjects, where *p* < 0.05 indicated significant departure. In line with our pre-specified inclusion criteria, the outcome measure in our study was the association between COL12A1 polymorphism and risk of ACLR, which could be either provided by the original study or calculated using the genotype distribution in ACLR and control group. We applied a “model-free approach” to determine the best-matching genetic model (Thakkinstian et al., 2005). Provided that the effecting allele was a and the wild type allele was A, then OR1, OR2, and OR3 were calculated for genotypes aa vs. AA, Aa vs. AA, and aa vs. Aa to capture the genetic effect of rs970547 on the risk of ACLR. The most appropriate genetic model was identified based on the relationships between the following three pairwise comparisons:

(1) Recessive model: if OR1 = OR3 ≠ 1 and OR2 = 1;
(2) Dominant model: if OR1 = OR2 ≠ 1 and OR3 = 1;
(3) Complete over-dominant model: if OR1 = 1, OR2 = 1/OR3 ≠ 1;
(4) Codominant model: if OR1 > OR2 > 1 and OR1 > OR3 > 1 (or OR1 < OR2 <1 and OR1 < OR3 <1).

In case that no appropriate genetic model could be found, the allele model was used to illustrate the results of our meta-analyses. The intra-study heterogeneity was evaluated using *Q*-statistical test and *I*^2^ test, where *p* < 0.1 and *I*^2^ > 50% indicated statistically significant heterogeneity (Higgins et al., 2003). However, regardless of the magnitude of inconsistency across studies, we used the random-effect model to combine data, due to the anticipated presence of heterogeneity (Mantel and Haenszel, 1959). Subgroup analysis by ethnicity and sex was conducted to test the genetic effect of rs970547 in different ethnicities and sexes. Funnel plot was generated using RevMan 5.3 software and then inspected for symmetry (Copenhagen: The Nordic Cochrane Centre, The Cochrane Collaboration, 2014). Egger's regression test and Begg's rank correlation test were also employed to evaluate the potential publication bias (Stata version 12.0, Stata Corp LP, USA) (Egger et al., 1997). The leave-one-out sensitivity analysis was carried out by removing each study at a time and re-evaluating the resulting effect on the overall effect. Sensitivity analysis was also performed by removing studies that did not conform to HWE and re-evaluating the resulting effect on the overall effect.

In order to evaluate whether our included studies had an adequate sample size to detect 30% increased or decreased risk of ACLR, we averaged the minor allele frequency (MAF) among control subjects. It turned out that the MAF among Asians and Caucasians were 38.13 and 22.54%, respectively. Therefore, at least 294 (147 cases and 147 controls) and 658 (329 cases and 329 controls) subjects were required for studies among Asians and Caucasians, respectively, to detect the association between the rs970547 polymorphism and susceptibility to ACLR, with a level of significance of 0.05 and power of 0.8 (https://clincalc.com/Stats/SampleSize.aspx).

## Results

### Literature Search

**Figure 2:**
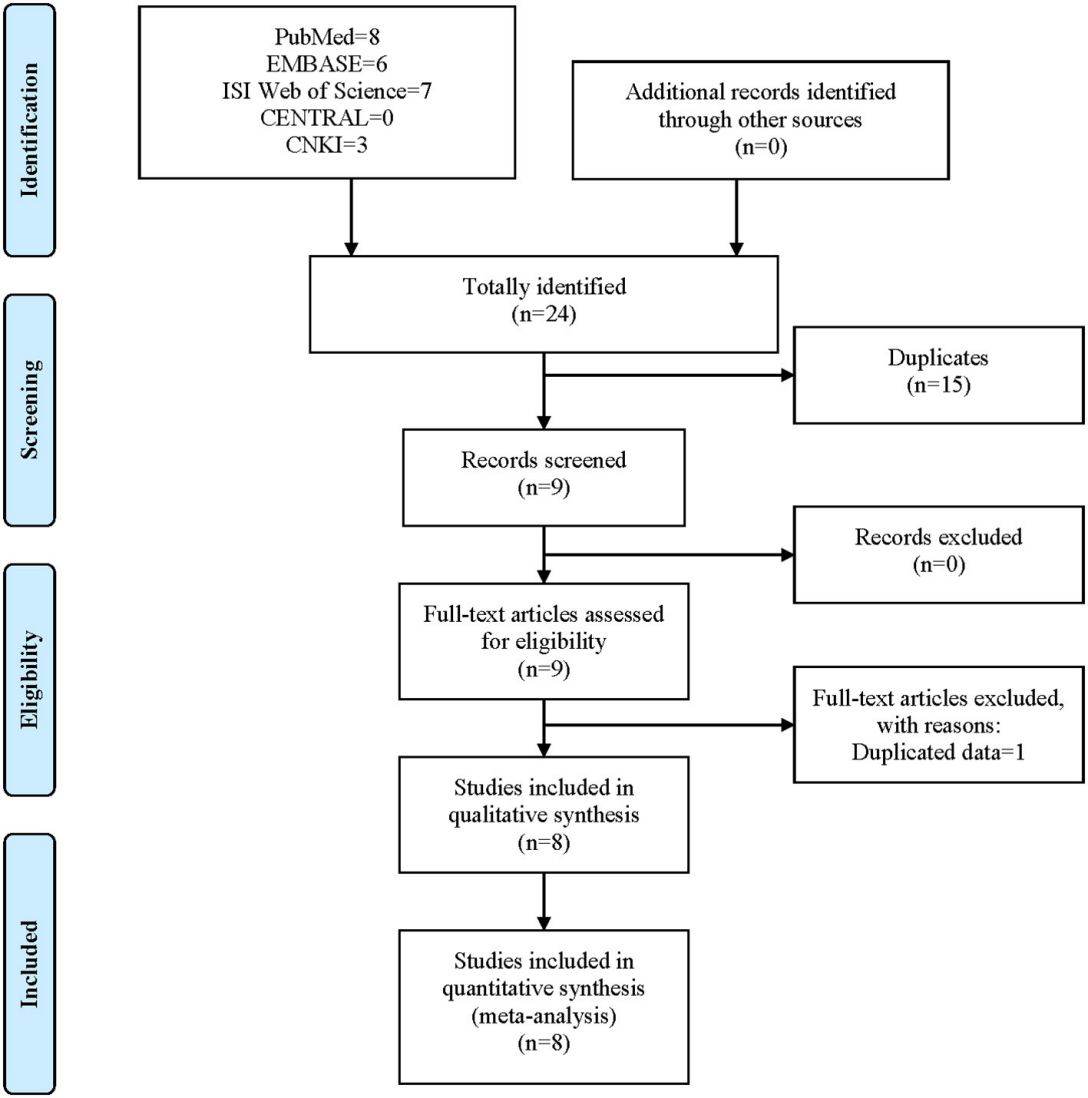
Flow chart of the literature search and screen.

The process of literature search and screen is shown in Figure 2. A total of 24 studies were identified after the initial literature search, namely, eight from PubMed, six from EMBASE, seven from ISI Web of Science, 0 from CENTRAL, and three from CNKI. No records were identified using other sources. After the removal of 15 duplicated records, nine studies (Posthumus et al., 2010; Ficek et al., 2014; O'Connell et al., 2015; John et al., 2016b; Chen et al., 2017; Kim et al., 2017; Kang et al., 2019; Sivertsen et al., 2019; Zhao et al., 2020) entered the first stage scanning of titles and abstracts. One study was further excluded due to duplicated data (Chen et al., 2017). Finally, eight studies (Posthumus et al., 2010; Ficek et al., 2014; O'Connell et al., 2015; John et al., 2016b; Kim et al., 2017; Kang et al., 2019; Sivertsen et al., 2019; Zhao et al., 2020) were deemed eligible for inclusion in our study, all of which were subsequently incorporated into the meta-analysis. It should be noted that one study contained two different cohorts (O'Connell et al., 2015).

### Main Characteristics

The main characteristics and methodological quality assessment of included studies are summarized in Table 1. Eight studies involving 1,477 patients with ACLR and 100,439 healthy controls were included in our current meta-analysis. All the studies were written in English and published between 2010 and 2020, with the cohort size ranging from 102 to 99,342. The majority of studies focused on the ACLR risk and COL12A1 polymorphism among Asians and Caucasians, while Kim and colleagues (Kim et al., 2017) enrolled a mixed population of Europeans, Latin Americans, and East Asians. Distinct techniques were also employed by our included studies for genotyping, including TaqMan, polymerase chain reaction-restricted fragment length polymorphisms (PCR-RFLP), SNaP-shot, and Affymetrix Axiom. On the basis of the NOS, each study received no less than six stars for methodological quality assessment, achieving an average of 6.5 stars (Table 2).

**Table 1:** Main characteristics of included studies.

**Study**	**Country**	**Ethnicity**	**Sample size (ACLR/control)**	**Genotyping method**	**ACLR**			**Control**			**HWE**
					**WT**	**HT**	**MT**	**WT**	**HT**	**MT**	
Ficek et al. (2014)	Poland	Caucasian	91/143	TaqMan	61	27	3	89	47	7	0.970
John et al. (2016)	India	Asian	50/52	PCR-RFLP	15	19	10	21	26	3	0.387
Kang et al. (2019)	South Korea	Asian	80/73	SNaPshot	41	31	8	22	40	11	0.584
Kim et al. (2017)	USA	Mixed	598/98744	Affymetrix Axiom	NR	NR	NR	NR	NR	NR	>0.05
O'Connell et al. (2015)	South Africa	Caucasian	218/226	TaqMan	140	65	13	133	87	6	0.170
O'Connell et al. (2015)	Poland	Caucasian	91/143	TaqMan	61	27	3	89	47	7	0.970
Posthumus et al. (2010)	South Africa	Caucasian	129/216	TaqMan	85	38	4	121	86	5	0.071
Sivertsen et al. (2019)	Finland	Caucasian	119/732	TaqMan	60	51	8	410	271	51	0.795
Zhao et al. (2020)	China	Asian	101/110	PCR-RFLP	48	42	11	39	59	16	0.701

*ACLR, anterior cruciate ligament rupture; WT/HT/MT, genotype counts of wild type, heterozygote, and mutant; HWE, Hardy-Weinberg Equilibrium; PCR-RFLP, polymerase chain reaction-restriction fragment length polymorphism; NR, not reported*.

**Table 2:** Quality assessment of included studies.

**Item/Study**	**Ficek et al. (2014)**	**John (2016)**	**Kang et al. (2019)**	**Kim et al. (2017)**	**O'Connell et al. (2015)**	**Posthumus et al. (2010)**	**Sivertsen et al. (2019)**	**Zhao et al. (2020)**
Adequate definition of cases	*	*	*	*	*	*	*	*
Representativeness of cases	-	-	-	-	-	-	-	-
Selection of control subjects	-	-	-	*	-	-	-	*
Definition of control subjects	*	*	*	*	*	*	*	*
Control for important factor or additional factor	**	**	*	**	*	*	*	*
Exposure assessment	*	*	*	*	*	*	*	*
Same method of ascertainment for all subjects	*	*	*	*	*	*	*	*
Non-response rate	*	*	*	*	*	*	*	*

*A study could be awarded a maximum of one star for each item except for the item “Control for important factor or additional factor”.*

*The definition/explanation of each column of the Newcastle-Ottawa Scale is available from http://www.ohri.ca/programs/clinical_epidemiology/oxford.asp*.

### Meta-Analysis Results

**Figure 3:**
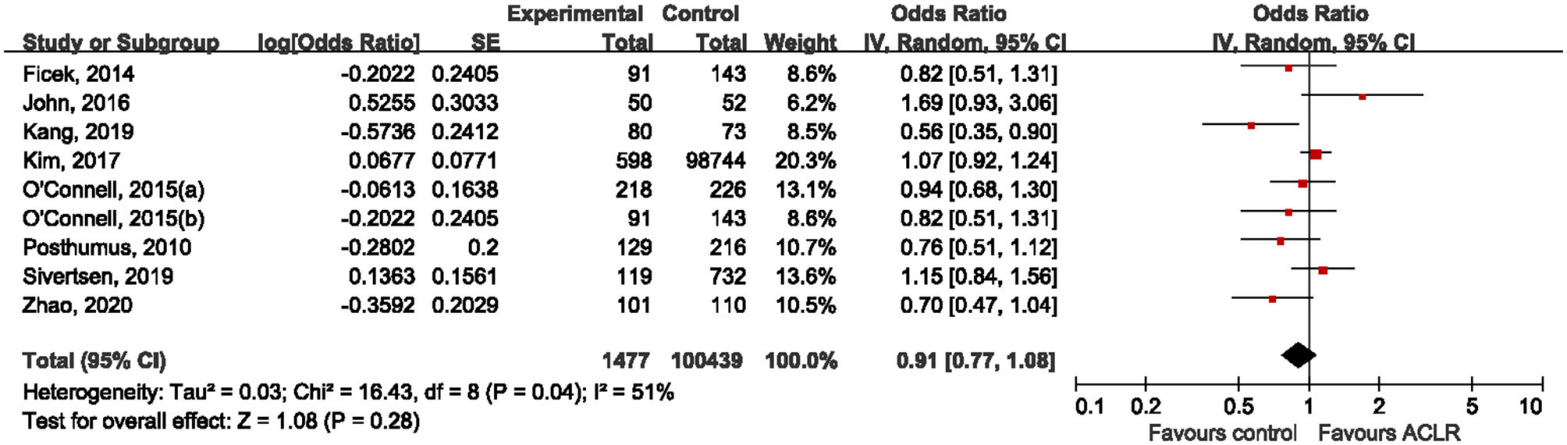
Forest plot of the association between rs970547 polymorphism in COL12A1 gene and risk of ACLR under the allele model (A vs. G).

Prior to combining data from each individual study, we applied a “model-free approach” to estimate the genetic effect of rs970547 on risk of ACLR. Seven studies (Posthumus et al., 2010; Ficek et al., 2014; O'Connell et al., 2015; John et al., 2016b; Kang et al., 2019; Sivertsen et al., 2019; Zhao et al., 2020) including eight independent cohorts provided detailed genotype counts in both the ACLR group and the control group, and therefore OR1, OR2, and OR3 were calculated to capture the magnitude of genetic effect of rs970547 among these seven studies. The estimated OR1 (aa vs. AA: 0.96, 95% CI 0.57–1.60; *p* = 0.86) and OR3 (aa vs. Aa: 1.26, 95% CI 0.81–1.94; *p* = 0.30) was nonsignificant while only OR2 (Aa vs. AA: 0.77, 95% CI 0.60–0.98; *p* = 0.03) was marginally significant. Based on the relationships between these three pairwise comparisons, rs970547 was not significantly associated with risk of ACLR. Therefore, we decided to illustrate the meta-analysis results using the allele model. As shown in Figure 3, the rs970547 was not associated with risk of ACLR (OR: 0.91, 95% CI 0.77, 1.08; *p* = 0.28) when using the allele model, and statistically significant heterogeneity was observed (*I*^2^ = 51%, *p* = 0.04). Random-effect model was used due to anticipated presence of heterogeneity.

### Subgroup Analysis and Publication Bias

**Figure 4:**
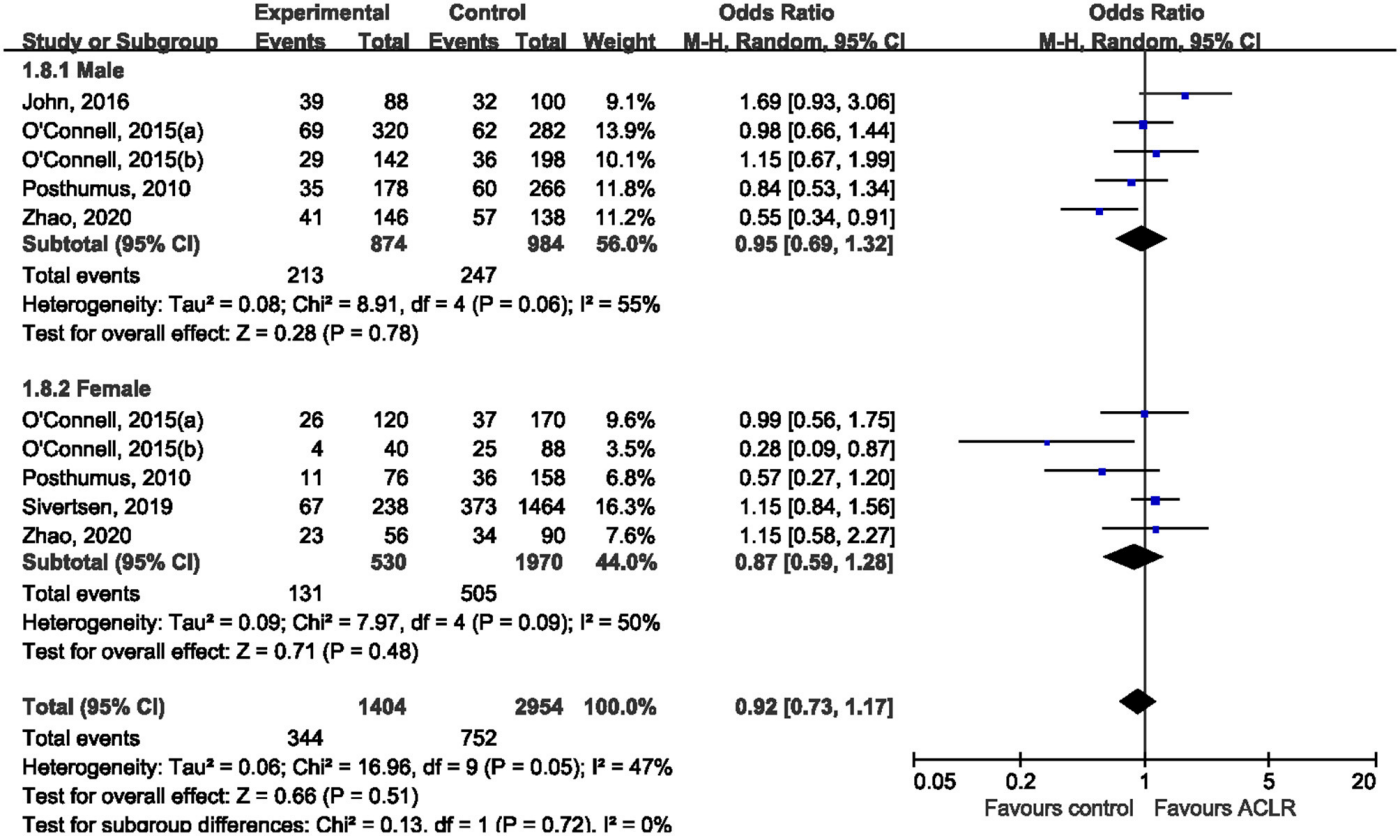
Stratification analyses of sex between rs970547 polymorphism in COL12A1 gene and ACLR risk under the allele model (A vs. G).

**Figure 5:**
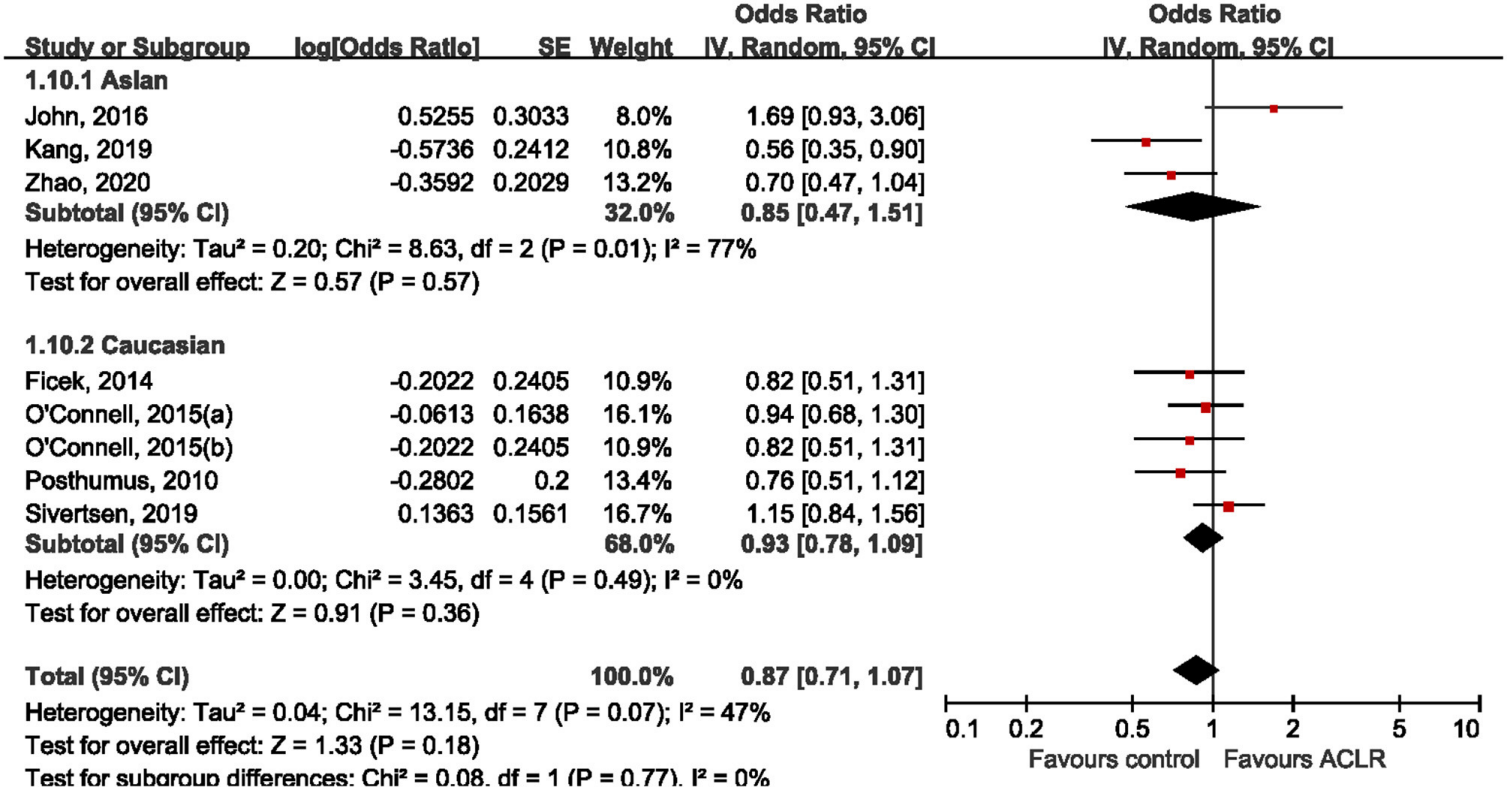
Stratification analyses of ethnicity between rs970547 polymorphism in COL12A1 gene and ACLR risk under the allele model (A vs. G).

Subgroup analysis was performed according to ethnicity and sex. Five studies (Posthumus et al., 2010; O'Connell et al., 2015; John et al., 2016b; Sivertsen et al., 2019; Zhao et al., 2020) reported genotype distribution among males and females, making it possible to conduct a stratified analysis by sex. Among male subjects, only one study (Zhao et al., 2020) suggested the significant association between rs970547 and risk of ACLR, while all the other included studies found null association (Figure 4). Pooled data showed that there was no significant association between rs970547 and ACLR risk among male subjects (OR: 0.95, 95% CI 0.69, 1.32; *p* = 0.78). Similarly, no association was found among females (OR: 0.87, 95% CI 0.59, 1.28; *p* = 0.48). Subgroup analysis by ethnicity was also performed to test the genetic effect of rs970547 in different populations. No association of rs970547 and ACLR risk could be found among either Asians (OR: 0.85, 95% CI 0.47, 1.51; *p* = 0.57) or Caucasians (OR: 0.93, 95% CI 0.78, 1.09; *p* = 0.36), as shown in Figure 5.

**Figure 6:**
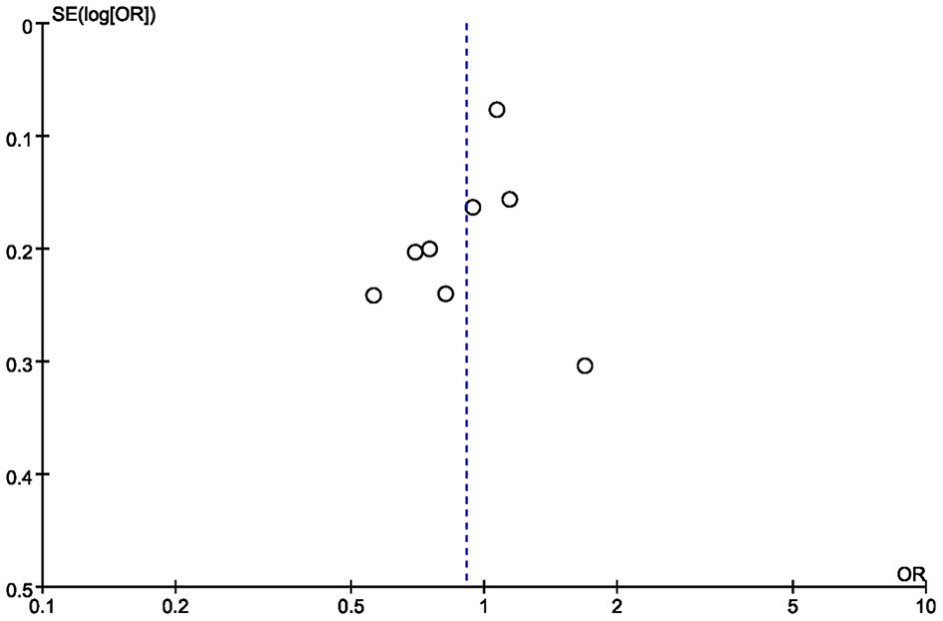
Funnel plot of the association between rs970547 polymorphism in COL12A1 gene and risk of ACLR.

The robustness of our conclusion was further confirmed by the leave-one-out sensitivity analysis (detailed data not shown). It should be noted that, after the removal of Kang's study, the intra-study heterogeneity diminished from moderate (*p* = 0.04, *I*^2^ = 51%) to low (*p* = 0.13, *I*^2^ = 37%). The funnel plot (Figure 6) was visually symmetrical, and the results of Begg's test (*z* = 1.04, *p* = 0.297) and Egger's test (*t* = −1.34, *p* = 0.222) also suggested no statistically significant publication bias.

## Discussion

As an intrinsic factor, the role of genetics in research of sports injury increases with every passing year, especially the genes encoding collagens that are the main component of ligaments. In our study, we included eight studies involving 1,477 patients with ACLR and 100,439 healthy controls and found that rs970547 was not associated with ACLR risk in the overall population. Furthermore, stratification analyses by ethnicity and sex indicated that rs970547 was not correlated with ACLR risk in female or male population, and Asian or Caucasian population. Our conclusion contradicted the previous meta-analysis by Lyu et al. (2018). In that meta-analysis, they reported a significant association between rs970547 and risk of ACLR, but only four case–control studies involving 603 patients and 789 healthy controls were included (Posthumus et al., 2010; Ficek et al., 2014; O'Connell et al., 2015; John et al., 2016b). The drawn conclusion could possibly be biased due to limited number of included studies and inadequate sample size. In contrast, our meta-analysis included eight studies with 1,477 patients with ACLR and 100,439 healthy controls, which significantly increased the power to detect the association between rs970547 and risk of ACLR. Furthermore, we adopted a “model-free” approach to avoid an inflated type I error.

As we know, ACLR represents one of the most frequent and severe injuries in major sports, and the quality of life of patients will be seriously impaired once ACLR occurs (Griffin et al., 2006; Beynnon et al., 2014). Even after ACL reconstruction, the knee pain, recreational limitations, and impaired quality of life will also be persistent. Additionally, an alarming number of individuals with ACLR will develop symptomatic knee osteoarthritis during young or middle adulthood. Due to the high incidence of rupture, high economic burden for individual and society, and unsatisfied clinical consequence, it is critical to clarify the underlying causes.

As a critical element, a number of studies have been performed to investigate the associations between COL12A1 gene polymorphisms and ACLR risk among different populations. The rs970547 polymorphism is located within the terminal exon 65 of COL12A1 gene, which changes the amino acid at position 3058 from a serine to glycine (September et al., 2008). As early as 2010, Posthumus and his colleagues investigated whether the rs970547 polymorphism was associated with ACLR in South African participants for the first time and found that the rs970547 polymorphism was significantly associated with the occurrence of ACLR in female and not in male and the whole population (Posthumus et al., 2010). Subsequent investigators conducted similar case–control genetic association studies in an Asian or Caucasian population, but studies did not yield consistent results. In their studies, Ficek et al. (2014) and O'Connell et al. (2015) basically concluded similar results to Posthumus et al., that the AA genotype of rs970547 was associated with increased risk of ACLR in female, but not male participants or the whole population, and it was worth noting that the main subjects of these studies were Caucasians. For the remaining studies, the conclusions were contradictory to the above studies. The results from John et al. (2016b) and Kang et al. (2019) showed that both the AA genotype and frequency of the A allele were at significantly higher risk of ACLR in the whole population. Furthermore, in a recent study, the authors demonstrated that male individuals carrying the COL12A1 gene rs970547 A allele and AA genotype may have an increased risk of ACLR (Zhao et al., 2020). In our meta-analysis, the combined data confirmed that the rs970547 polymorphism was not related to the risk of ACLR, and in stratification analyses by ethnicity or sex, this conclusion was confirmed once again.

The rs970547 polymorphism is a non-synonymous coding variant causing amino acid at position 3058 to change from serine to glycine. Because the wild-type serine amino acid is a neutral polar amino acid with a larger side chain than the substituted non-polar neutral glycine amino acid, some investigators speculate that the amino acid replacement may alter the biomechanical properties of the collagen fibril and affect the occurrence of ACLR. However, till now, no conclusive evidence could be found to suggest the functional consequence of this change in amino acid sequence. We also searched the GTEx Portal (https:/gtexportal.org/ accessed on July 1, 2021) and found no data to support the association between rs970547 and altered gene expression in different human tissues. The results of our current study also suggested no association between rs970547 and risk of ACLR. In our included studies, the male and female in control and ACLR groups were not similarly matched in physical activity. Briefly, the female in the ACLR group had significantly longer period of non-contact jumping sports while the male had longer history of contact sports (Posthumus et al., 2010). The participants in the study conducted by Ficek et al. are professional soccer players, whereas other studies included only recreational athletes. In addition, a number of intrinsic risk factors classified as either anatomical, hormonal, or neuromuscular cannot be ruled out; it remained possible that even though the biomechanical properties of the ACL were influenced by the rs970547 polymorphism in male and female participants, it did not alter the risk of ACL ruptures in the male participants significantly enough to demonstrate an association in our all-included study. As a matter of fact, our conclusion was consistent with the results of a previous study to determine the effect of rs970547 in Achilles tendon injury, in which no statistically significant differences were identified in the genotype, allele, or haplotype distributions between the affected and control subjects (September et al., 2008).

Several limitations to our study should be acknowledged when interpreting the results. Firstly, the sample size of each individual study was not adequate enough to detect the genetic association between rs970547 and risk of ACLR. In our meta-analysis, the majority of included studies enrolled several hundreds of participants except one study with large-scale genotype and phenotype data. However, it should be noted that the pooled analysis had an adequate sample size according to the sample size calculation (294 for Asians and 658 for Caucasians). Secondly, although we performed stratification analyses by ethnicity or sex, we were unable to rule out the intrinsic and extrinsic factors mixed in study. For instance, the current body of evidence did not allow us to perform a subgroup analysis of injury mechanisms (contact or non-contact mechanism). Thirdly, majority of our included studies were conducted in a Caucasian population. Only three studies were designed to investigate the genetic effect of COL12A1 polymorphisms in an Asian population, and the sample size was limited. More high-quality studies within different ethnic backgrounds are needed to verify the genetic effect of COL12A1 polymorphisms in risk of ACLR. If the effect of rs970547 in pathogenesis of ACLR could be confirmed by future studies, it would be of great interest to dive deeper into the possible link of COL12A1 polymorphisms and other types of other musculoskeletal soft-tissue injuries, such as Achilles tendon injuries and rotator cuff injuries, to facilitate our understanding of the biomechanical properties of tendons at a molecular level as well as ligament and tendon injuries that occur during physical activities.

Taken together, the findings of this current meta-analysis suggested that rs970547 was not associated with ACLR risk in male, female, and the overall population among Asians or Caucasians. However, larger-scale studies within different ethnic backgrounds are still warranted to confirm the findings of our study.

## Data Availability Statement

The original contributions presented in the study are included in the article/supplementary material, further inquiries can be directed to the corresponding authors.

## Author Contributions

J-mH and Z-tL conceived and devised the study. J-mH, Z-tL, and D-mZ performed the experiments and wrote the paper. WW and D-mZ analyzed the data. WW and Z-tL revised the manuscript. All authors have contributed to the final version and approved the publication of the final manuscript.

## Conflict of Interest

The authors declare that the research was conducted in the absence of any commercial or financial relationships that could be construed as a potential conflict of interest.

## Publisher's Note

All claims expressed in this article are solely those of the authors and do not necessarily represent those of their affiliated organizations, or those of the publisher, the editors and the reviewers. Any product that may be evaluated in this article, or claim that may be made by its manufacturer, is not guaranteed or endorsed by the publisher.
